# Detection of early stage ovarian cancer in a large community cohort

**DOI:** 10.1002/cam4.2522

**Published:** 2019-09-30

**Authors:** Elizabeth J. Suh‐Burgmann, Mubarika Alavi

**Affiliations:** ^1^ Division of Gynecologic Oncology The Permanente Medical Group Walnut Creek California; ^2^ Division of Research Kaiser Permanente Northern California Oakland California

**Keywords:** early detection of cancer, ovarian cancer, ovarian neoplasms

## Abstract

**Background:**

Although detecting ovarian cancer at early stage is a highly meaningful clinical goal, no studies have evaluated early stage disease presentation in a large community‐based population and how it differs from that of late stage disease.

**Methods:**

Electronic medical records were evaluated for women diagnosed with ovarian or fallopian tube cancer in 2016 and 2017 to identify the first imaging study to detect disease. Women being followed prior to diagnosis for known genetic risk from BRCA or other mutation were excluded. The visit in which the imaging test was ordered and related encounters were reviewed to determine the indication for imaging. Patient characteristics, presenting symptoms and duration, and modality of first abnormal imaging were compared for early vs late stage ovarian cancer and by provider specialty.

**Results:**

Of 540 women with ovarian cancer, 190 (35%) were diagnosed with early stage disease, of whom 141 (74%) were symptomatic, with 45% of women presenting to internists, 33% to gynecologists, and 20% to emergency medicine physicians. Pelvic ultrasonography detected only 23% of late stage cases whereas pelvic ultrasonography and abdominal pelvic computed tomography (CT) each detected 47% of early stage cases. While abdominal pain and bloating were common to both women with early and late stage cancer, women with early stage disease were younger (58 vs 64 years, *P* < .0001), more likely to present to gynecologists (33% vs 15%, *P* < .001) and complained more often of a palpable mass (17% vs 6%, *P* < .0001) or postmenopausal bleeding (11% vs 5%, *P* < .001).

**Conclusions:**

Excluding women with genetic predisposition to ovarian cancer known prior to diagnosis, approximately three out of four cases of early stage ovarian cancer are detected as the result of evaluation of symptoms and one in four cases are detected incidentally. Abdominal pelvic CT and pelvic ultrasonography each detect an equal proportion of early stage cases. In contrast to late stage presentation, women diagnosed with early stage disease present more often with complaints of a palpable mass or postmenopausal bleeding, particularly to gynecologists.

## Graphical abstract

In a large community cohort, 35% of ovarian cancers were diagnosed at early stage. While nongynecologists detect the majority of both early and late stage cases, gynecologists detect 1 in 3 early stage cancers, which, compared with late stage cancer, are more likely to be associated with presenting complaints of a palpable mass or postmenopausal bleeding.

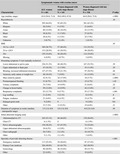

## 1 INTRODUCTION

Ovarian cancer affects approximately 22 000 women annually in the US and ranks 11th in numbers of women affected but 5th in cancer deaths, largely due to frequent diagnosis at late stage.^1^ Randomized controlled screening trials using CA 125 tumor marker testing and ultrasonography have failed to demonstrate survival benefit in screened compared to unscreened groups resulting in a recommendation against screening by the American Cancer Society and the US Preventive Services Task Force (USPTF).^2^, ^3^ In the absence of effective screening, “appropriate clinical suspicion” of the signs and symptoms of disease is currently the best strategy to detect ovarian cancer, according to the American College of Obstetrics and Gynecology.^4^ The hypothesis that ovarian cancer outcomes could be improved through better symptom recognition has been largely advanced by Goff et al who proposed a “symptom index” as a tool to screen women for further evaluation for ovarian cancer; the index is considered positive if pelvic or abdominal pain, increased abdominal size, bloating, difficulty eating, or early satiety occur greater than 12 times per month but for less than 1 year.^5^, ^6^ However, since the main determinant of ovarian cancer prognosis is stage at diagnosis, symptom recognition must enable detection of early stage disease to effect survival. Ovarian cancer symptoms have largely been defined by patient recall in case‐control studies,^7^, ^8^, ^9^, ^10^, ^11^, ^12^, ^13^, ^14^ which include relatively few patients with early stage invasive cancer. No studies have described early ovarian cancer presentation for an entire community‐based population to inform how it differs from that of late stage disease and where opportunities to detect early stage disease are most likely to be found in clinical practice. We therefore evaluated the clinical presentation of early vs late stage ovarian cancer and the role of different specialties in disease detection for a large unselected population of women diagnosed with ovarian cancer.

We conducted a retrospective cohort study of all women aged 18 or older, diagnosed with primary ovarian or fallopian tube cancer in 2016 and 2017. Participants were members of Kaiser Permanente Northern California, an integrated healthcare delivery system, including 21 hospitals, 46 facilities and more than 8000 physicians, that provides care for over 4.2 million members annually. The ethnic and racial diversity of health plan membership mirrors those of the geographic areas served, with 47% Caucasian, 7% African‐American, 22% Hispanic, 20% Asian or Pacific Islander, and 3% multiracial. Approval for the study was obtained from the Kaiser Permanente Northern California Institutional Review Board. During the study period, the health system included approximately 600 women's health providers (henceforth referred to as “gynecologists”), approximately 4500 adult primary care medicine providers (henceforth referred to as “internists”), and approximately 800 emergency medicine physicians. Access to both gynecologists and internists is unrestricted in the health system and does not require referral.

All cases of primary ovarian or fallopian tube cancer in 2016 and 2017 were identified by the institution's comprehensive tumor registry, which reports to the California and Surveillance, Epidemiology and End Results Cancer Registries. Internal quality studies indicate the registry detects >98% of all cancers within health plan members. Women under age 18, at known elevated genetic risk of ovarian cancer due to positive BRCA carrier status or other genetic assessment prior to cancer diagnosis or a prior history of ovarian cancer, those diagnosed with borderline tumors (including those with microinvasion and non‐invasive metastases) or missing information due to the diagnosis being established outside the health system were excluded. Stage was determined by surgical pathology findings and classified as early (Stage I‐II), or late (Stage III‐IV) based, based on AJCC 7th surgical‐pathological staging classification schema.^15^ In cases treated with neoadjuvant chemotherapy or in women who declined treatment, late stage was assumed if imaging revealed obvious extra‐pelvic or nodal metastatic disease—otherwise, stage was considered indeterminate.

For each woman, all radiologic and nuclear medicine imaging studies done within 2 years prior to diagnosis were reviewed to identify the first imaging study to detect disease by reporting one of the following: abnormal adnexal or ovarian cyst or mass, abnormal abdominal or pelvic mass, abdominal or pelvic adenopathy, carcinomatosis, ascites or pleural effusion. We defined as presenting complaints those that led to the imaging test, determined from chart review of the clinical encounter in which the imaging test was ordered and any preceding encounters related to the same complaints, and estimated based on this the duration of symptoms at the time imaging was ordered. Symptoms were classified as (a) lower abdominal pain/pelvic pain; (b) upper abdominal/flank pain; (c) change in bowel habits (constipation, diarrhea, symptoms related to bowel movements); (d) urinary (frequency, urgency, hematuria); (e) bloating/increased abdominal distension; (f) anorexia/early satiety/weight loss; (g) respiratory (persistent cough, shortness of breath or dyspnea on exertion); (h) fatigue/weakness; (i) leg edema; (j) groin node or 11) other. Duration of symptoms leading to imaging was recorded in weeks with a minimum of 1 and a maximum of 52 weeks. For patients who were asymptomatic, the circumstances leading to the diagnosis were assessed. We considered women with disease unexpectedly found at surgery for another indication to be asymptomatic as it was not possible in these cases to reliably distinguish preoperative symptoms specific to ovarian cancer.

The provider ordering the initial abnormal imaging test was considered the provider that detected disease unless symptoms were first evaluated by an outpatient provider who then transferred the patient to the emergency department where imaging was performed, in which case the outpatient provider was considered the detecting provider. Emergency medicine providers were only considered the detecting provider for women who self‐referred to the emergency department. The specialty of the provider was determined from medical group databases.

Patient characteristics, presenting complaints, duration of symptoms, and modality of imaging to first detect disease were analyzed in comparisons by stage (early vs late), as well as by provider specialty.

Statistical analysis for continuous variables was performed using the Kruskal‐Wallis Test which compares two or more independent samples of equal or different sample sizes. The categorical variables were assessed using *χ* ^2^ tests for homogeneity or independence. For sparse data we used the Fisher's exact test. Analyses were performed using SAS software, version 9.4 (SAS Institute Inc), with a *P* value of <.05 for statistical significance.

## 2 RESULTS

**Figure 1:**
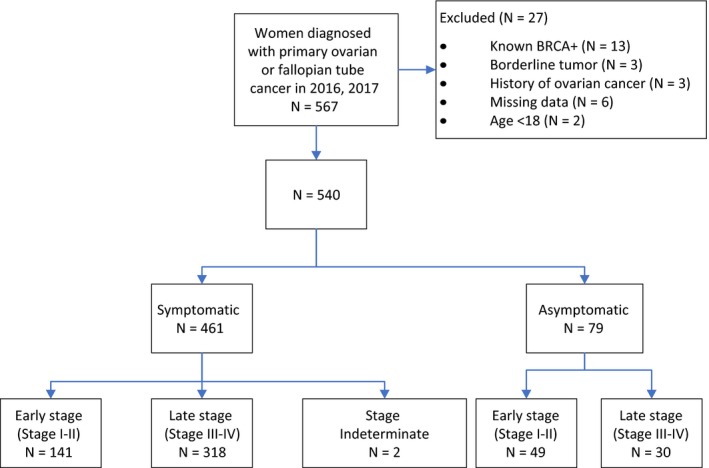
Flowchart of cohort

Among 567 women diagnosed with ovarian or fallopian tube cancer (293 in 2016 and 274 in 2017), 27 (5%) were excluded. A flowchart of the exclusions applied to the cohort is shown in Figure 1. For efficiency, women with both ovarian or fallopian tube cancer are referred to as having ovarian cancer.

Of the remaining 540 women, 79 (15%) were asymptomatic with disease incidentally detected, with 49 (62%) diagnosed with early stage and 30 (38%) with late stage disease. Of the 461 (85%) of women who were symptomatic prior to diagnosis, 141 (31%) were diagnosed with early and 318 (69%) with late stage cancer, with stage considered indeterminant for 2 women who had apparent early stage disease on imaging but did not undergo surgical evaluation due to advanced age and medical morbidity. Patient characteristics, presenting symptoms, initial abnormal imaging modality, and specialty of the detecting provider of symptomatic women with early and late stage disease is shown in Table 1. Overall, 49% of ovarian cancer cases were detected by internists, 21% by gynecologists, 25% by emergency medicine physicians, and 5% by other providers. Women with early stage disease were younger (median age 58 vs 64 years, *P* < .0001), more likely to present to gynecologists (33% vs 16%, *P* = .0003) and to have their disease first detected by pelvic ultrasonography (48% vs 23%, *P* < .0001). Lower abdominal pain was a presenting complaint in a similar proportion of women with early and late stage disease (48% vs 52%), but women with early stage disease were less likely to present with anorexia (5% vs 13%, *P* = .01), or respiratory complaints (6% vs 17%, *P* = .0008), and more likely to present with postmenopausal bleeding (11% vs 5%, *P* = .009) or complain of feeling a mass (17% vs 6%, *P* < .0001). Bloating was also less common among women with early stage cancer (21% vs 31%, *P* = .04) but still common relative to other symptoms. Overall, the 4 most common presenting symptoms associated with early stage disease were lower abdominal pain (48%), bloating (21%), a mass (17%) and postmenopausal bleeding (11%) which together identified 84% of early stage cases. The median duration of any symptoms among women at the time of imaging was shorter for women diagnosed with early compared to late stage disease (2 vs 4 weeks, *P* = .04).

**Table 1:** Characteristics, symptoms, and initial abnormal imaging for women with early vs late stage ovarian cancer

Characteristic	Symptomatic women with ovarian cancer			*P*‐value
	ALL women N = 461	Women diagnosed with early stage disease N = 141	Women diagnosed with late stage disease N = 318	
Age (median, range)	63.0 (54.0, 71.0)	58.0 (49.0, 67.0)	64.0 (56.0, 73.0)	<.0001
Race/ethnicity				.16
White	295 (64.0%)	92 (65.2%)	201 (63.2%)	
Hispanic	26 (5.6%)	5 (3.5%)	21 (6.6%)	
Asian	69 (15.0%)	26 (18.4%)	43 (13.5%)	
Black	38 (8.2%)	11 (7.8%)	27 (8.5%)	
Other	30 (6.5%)	5 (3.5%)	25 (7.9%)	
Unknown	3 (0.7%)	2 (1.4%)	1 (0.3%)	
BMI				.45
18.5 to <24.9	169 (36.7%)	57 (40.4%)	112 (35.2%)	
25 to <29.9	133 (28.9%)	43 (30.5%)	89 (28.0%)	
30+	155 (33.6%)	40 (28.4%)	114 (35.8%)	
Missing	4 (0.9%)	1 (0.7%)	3 (0.9%)	
Presenting symptoms % (not mutually exclusive)				
Lower abdominal or pelvic pain	236 (51.2%)	68 (48.2%)	167 (52.5%)	.39
Upper abdominal or flank pain	47 (10.2%)	11 (7.8%)	35 (11.0%)	.29
Bloating, increased abdominal distention	127 (27.5%)	30 (21.3%)	97 (30.5%)	.04
Anorexia, early satiety or weight loss	48 (10.4%)	7 (5.0%)	41 (12.9%)	.01
Mass noted by patient	42 (9.1%)	24 (17.0%)	18 (5.7%)	<.0001
Postmenopausal bleeding	31 (6.7%)	16 (11.3%)	15 (4.7%)	<.01
Urinary complaints	35 (7.6%)	14 (9.9%)	21 (6.6%)	.22
Change in bowel habits	59 (12.8%)	14 (9.9%)	44 (13.8%)	.25
Respiratory complaints	63 (13.7%)	8 (5.7%)	55 (17.3%)	<.001
Leg swelling	11 (2.4%)	3 (2.1%)	8 (2.5%)	.8
Fatigue, weakness	21 (4.6%)	6 (4.3%)	15 (4.7%)	.83
Enlarged groin node	9 (2.0%)	0	9 (2.8%)	NA
Other	33 (7.2%)	13 (9.2%)	19 (6.0%)	.21
Duration of symptoms in weeks (median, interquartile range)	3.5 (1.0, 8.0)	2.0 (1.0, 8.0)	4.0 (2.0, 8.0)	.04
Initial abnormal imaging study				<.0001
Abdominal/pelvic CT	245 (53.1%)	66 (46.8%)	178 (56.0%)	
Chest CT	6 (1.3%)	0	6 (1.9%)	
Pelvic ultrasonography	139 (30.2%)	67 (47.5%)	72 (22.6%)	
Upper abdominal ultrasonography	30 (6.5%)	4 (2.8%)	25 (7.9%)	
Chest radiograph	36 (7.8%)	2 (1.4%)	34 (10.7%)	
Other	5 (1.1%)	2 (1.4%)	3 (0.9%)	
Specialty of provider detecting disease				<.001
Emergency medicine	117 (25.4%)	29 (20.6%)	87 (27.4%)	
Primary Care medicine	226 (49.0%)	63 (44.7%)	162 (50.9%)	
Gynecology	96 (20.8%)	46 (32.6%)	50 (15.7%)	
Other	22 (4.8%)	3 (2.1%)	19 (6.0%)	

Abbreviation: CT, computed tomography.

To better inform early stage disease presentation from the perspective of clinicians, symptoms were compared for women with early stage disease by provider specialty (Table 2). Lower abdominal pain accounted for 76% of women presenting to emergency physicians but only 41% of women presenting to internists and gynecologists. After lower abdominal pain, the most common presenting symptoms to internists were bloating (29%), feeling a mass (17%), upper abdominal or flank pain (14%) and change in bowel habits (13%), whereas postmenopausal bleeding was reported by 28%, and a palpable mass by 24% of women presenting to gynecologists. Together, postmenopausal bleeding and a palpable mass identified 40% of women with early stage disease presenting to gynecologists, and 27% of all early stage cases.

**Table 2:** Presenting symptoms for women with early stage ovarian cancer by provider specialty

Characteristic	Symptomatic early stage ovarian cancer			*P*‐value
	Women with disease detected by gynecologists N = 46	Women with disease detected by primary care medicine N = 63	Women with disease detected by emergency medicine N = 29	
Age, y (median, range)	59.0 (47.0, 68.0)	58.0 (52.0, 66.0)	56.0 (43.0, 63.0)	.3
Presenting symptoms% (not mutually exclusive)				
Lower abdominal pain	19 (41.3%)	26 (41.3%)	22 (75.9%)	.004
Upper abdominal or flank pain	0	9 (14.3%)	2 (6.9%)	.01
Bloating, increased abdominal distention	8 (17.4%)	18 (28.6%)	4 (13.8%)	.19
Anorexia, early satiety or weight loss	1 (2.2%)	5 (7.9%)	1 (3.4%)	.54
Mass noted by patient	11 (23.9%)	11 (17.5%)	2 (6.9%)	.15
Postmenopausal bleeding	13 (28.3%)	3 (4.8%)	0	<.001
Urinary complaints	6 (13.0%)	7 (11.1%)	1 (3.4%)	.44
Change in bowel habits	2 (4.3%)	8 (12.7%)	3 (10.3%)	.35
Respiratory complaints	0	5 (7.9%)	3 (10.3%)	.07
Leg swelling	0	3 (4.8%)	0	.31
Fatigue, weakness	0	3 (4.8%)	3 (10.3%)	.07
Enlarged groin node	0	0	0	NA
Other	4 (8.7%)	4 (6.3%)	3 (10.3%)	.77
Initial abnormal imaging study				<.0001
Abdominal/pelvic CT	1 (2.2%)	42 (66.7%)	20 (69.0%)	
Chest CT	0	0	0	
Pelvic ultrasonography	44 (95.7%)	16 (25.4%)	7 (24.1%)	
Upper abdominal ultrasonography	0	3 (4.8%)	1 (3.4%)	
Chest radiograph	0	1 (1.6%)	1 (3.4%)	
Other	1 (2.2%)	1 (1.6%)	0	

Abbreviation: CT, computed tomography.

The circumstances leading to disease detection for 79 asymptomatic women and stage at diagnosis are summarized in Table 3, with incidental findings on imaging obtained for an unrelated concern being the most common scenario leading to detection of asymptomatic early stage disease. In addition, 19 women with early stage endometroid ovarian cancer were diagnosed as a dual primary cancer at surgery for known endometrial uterine cancer.

**Table 3:** Circumstances leading to diagnosis of ovarian cancer among asymptomatic women^a^

Disease detected by	All cancers	Early stage
Incidental finding on imaging for unrelated concern	22	11
Concurrent ovarian cancer at surgery for endometrial cancer	19	14
Incidental finding of mass on routine pelvic exam	11	9
Incidental finding at unrelated surgery (other than for endometrial cancer)	12	8
Incidental finding of mass on routine abdominal exam	6	3
Incidental finding on routine colonoscopy	4	2
Incidental finding on routine cervical cytology	3	0
Growth of adnexal mass on follow‐up ultrasound	2	2
Total	79	49

a Excludes women with BRCA mutation or other genetic predisposition known prior to diagnosis.

## 3 DISCUSSION

To the best of our knowledge, this is the first study to evaluate ovarian cancer clinical presentation for an entire large, diverse community‐based population including how presenting symptoms and the diagnostic process vary for early compared to late stage cancers and by clinical specialty. Overall, three of four women with ovarian cancer have disease detected by non‐gynecologists, with internists detecting 49% of ovarian cancers overall and 45% of early stage cases compared to gynecologists detecting 21% of cancers overall and 33% of early stage cases. Abdominal pelvic computed tomography (CT) scan was much more likely than pelvic ultrasonography to be the imaging that detected late stage disease (56% vs 22%), but pelvic ultrasonography was as likely as CT to detect early stage disease (47%). Interestingly, other imaging such as chest radiograph and upper abdominal ultrasonography were the first imaging indication of disease for 20% of late stage ovarian cancer cases, which is consistent with our finding that a significant proportion of women present with respiratory complaints (17%) or upper abdominal pain (11%), which are symptoms not typically thought to be associated with ovarian cancer.

Gynecologists detected a greater proportion of early compared to late stage disease (33% vs 16%, *P* < .0001). This was noted in a previous study although an explanation was not provided.^16^ We found this to be attributable to women with early stage cancer presenting more often with postmenopausal bleeding or a complaint of a mass. Together these two presenting complaints characterized 40% of the early stage cancer cases detected by gynecologists. Indeed, for every symptom, the majority of women with that symptom were diagnosed with late stage cancer, except for postmenopausal bleeding and a palpable mass, for which 52% and 57% of women, respectively, were diagnosed with early stage cancer. This suggests that these two symptoms are more likely to be true “early warning signs” of ovarian cancer. Consistent with previous studies,^5^, ^6^, ^7^, ^8^, ^9^, ^10^, ^11^, ^12^, ^13^ we found lower abdominal pain and bloating to be common presenting complaints of ovarian cancer and we observed similar frequencies of lower abdominal pain (48% vs 52%) but somewhat lower rates of bloating (21% vs 31%, *P* = .04) among women diagnosed with early compared to late stage disease. While the duration of symptoms was overall shorter for women diagnosed with early stage disease, the time difference observed is unlikely to account for difference in stage. The specific factors that cause some women to complain of pain and other symptoms at early stage when others appear to have only reported symptoms once they had late stage disease are unclear.

Twenty‐six percent of early ovarian cancers were diagnosed in asymptomatic women, despite exclusion of women known to be at elevated genetic risk prior to diagnosis. This included 9 women who were diagnosed as the result of an asymptomatic mass noted on routine pelvic exam. While the USPTF gives pelvic exams in primary care a grade of “I” (insufficient evidence) due to lack of data regarding their impact on mortality,^17^ our data indicate that routine pelvic exam led to detection of a significant proportion of women with early stage disease diagnosed by gynecologists. Only 2 (0.4%) ovarian cancers were detected solely due to an observed increase in ovarian cysts or masses on follow‐up ultrasound in the absence of symptoms or other stigmata of malignancy. While asymptomatic ovarian cysts are extremely common, often leading to additional imaging, anxiety and potentially unnecessary surgery, our findings underscore the rarity of finding malignancy among isolated asymptomatic ovarian cysts that are candidates for ultrasound monitoring.^18^

Key strengths of the study are the large number of women included with early stage invasive disease, the demographically diverse community‐based nature of the cohort, which avoids selection of certain cancer presentations over others and increases generalizability to different populations, the ability to evaluate clinical presentation by specialty, the exclusion of women whose diagnostic process was driven by known preexisting risk, and the high degree of capture of imaging and clinical encounters leading to diagnosis including emergency department visits, email messages and telephone calls. Women with early stage invasive disease have been underrepresented in most studies evaluating symptoms and, in older studies, patients with early stage disease often included women with borderline tumors, which have an overwhelmingly benign behavior and are now excluded from most cancer registries.^19^, ^20^ Previous studies also evaluated symptoms primarily by patient recall in surveys, which may bias identification of certain symptoms by the questions posed, may exclude some patients such as those with aggressive disease and may not accurately reflect the complaints patients present to providers. While patient recall may more completely capture symptoms, clinician vigilance may be better informed by patient complaints observed in actual practice.

In the healthcare system in which the study was conducted, women have unrestricted access to gynecologists as well as primary care medicine providers. The proportion of disease detected by various specialties may be different for women in non‐US practice settings in which providers have different scopes of practice or in populations with limited access to health care providers. While we observed that 25% of women had disease detected upon self‐referral to the emergency department despite their having access to outpatient care, it is likely that this proportion would be higher among populations with restricted access to non‐urgent medical care. Limitations of our study also include the potential for information bias in identifying presenting symptoms, defined as the symptoms that led to initial detection of disease by imaging. In some cases, women saw multiple providers for a variety of reasons during the months prior to diagnosis and it is possible that an encounter with another provider was also related to disease—for example a visit to primary care for cough a month prior to a visit with gynecology for pelvic pain may have also been related to disease but was not considered part of the disease presentation. The recorded duration of symptoms reflects only those symptoms that led to diagnostic imaging and may therefore underestimate the duration of all disease related symptoms. The thoroughness and accuracy of symptom documentation in the electronic medical record undoubtedly varied across providers. However, in all cases the reasons for ordering the initial abnormal diagnostic imaging study was documented in notes entered at the point of care, prior to the diagnosis being established.

In summary, excluding women with genetic predisposition to ovarian cancer known prior to diagnosis, approximately three out of four cases of early stage ovarian cancer are detected as the result of evaluation of symptoms and one in four cases are detected incidentally. In contrast to late stage presentation, women diagnosed with early stage disease present more often with complaints of a palpable mass or postmenopausal bleeding, particularly to gynecologists. These findings inform how clinicians are most likely to encounter ovarian cancer and may help guide strategies aimed at optimizing early detection through symptom recognition.

## CONFLICT OF INTEREST

None declared.

## AUTHOR CONTRIBUTIONS

Elizabeth Suh‐Burgmann involved in conceptualization, funding acquisition, manual chart review, data interpretation, and writing; Mubarika Alavi: data curation, formal analysis.

## Data Availability

The data that support the findings of this study are available on request from the corresponding author. The data are not publicly available due to privacy or ethical restrictions.
